# PotteryVR: virtual reality pottery

**DOI:** 10.1007/s00371-022-02521-2

**Published:** 2022-06-20

**Authors:** Sarah Dashti, Edmond Prakash, Andres Adolfo Navarro-Newball, Fiaz Hussain, Fiona Carroll

**Affiliations:** 1 Cardiff Metropolitan University, Cardiff, UK; 2 University for the Creative Arts, Farnham, UK; 3 Pontificia Universidad Javeriana, Cali, Colombia

**Keywords:** Virtual pottery, Usability, Evaluation, Interaction, Methods, Creative technology

## Abstract

Handcrafting ceramic pottery in the traditional method or virtual reality (VR) with intricate surface details is still challenging for the ceramic and graphic artist. Free-form pottery modeling can be efficiently geometrically modeled with the right tools with detailed 3D print outputs, yet challenging to be manufactured using traditional art. The new advanced pottery VR simulation is a promising method to recreate the traditional pottery simulation for a better experience with some barriers. The challenges that arise from surface detail in pottery are a tedious task accomplished by mesh blending and retopology. This paper focuses on refining the VP application’s performance by adding unique sound resonance as a more likely infinite geometric phenomenon textures, blending it into the basic shapes. This paper combines creativity and visual computing technologies such as VR, mesh blending, fixing errors, and 3D printing to bring the ceramic artist’s imagination to life. We have used sound resonance with virtual pottery (VP) systems refinements to demonstrate several standard pottery methods from free form deformed pottery, retopology, mesh blended for surface details, and 3D printed pottery with materials including polymer and ceramic resins.

## Introduction

### Creative interactions

VR has engaged with new and varied fields throughout the last decade. Creative interactions with more engagement between humans and machines empower science, art, and technology. The new technologies, such as advanced graphics engines, wearable devices, and innovative interface systems, open a new gateway across disciplines. The concept of VR modeling applications has changed with the growth beyond disciplinary fields, improving the human ability to unify physical and visual interaction and exploration [1].

### Traditional to virtual pottery

**Fig. 1:**
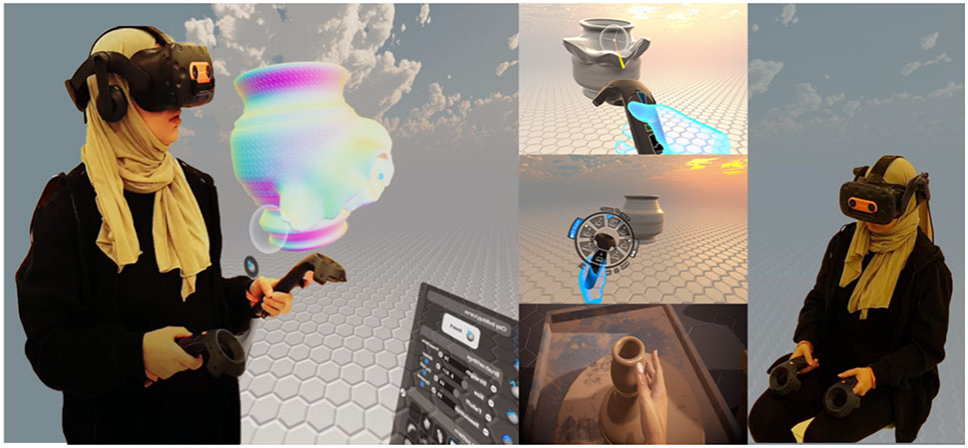
Usability of VP physical and visual interaction

It is a unique path of creating complex objects using VR clay deformation in virtually perceived experiences such as virtual pottery (VP) [2]. We have researched the most missing factor and how to implement it with existing tools. Those explorations increased our motivation to develop the missing aspects of VP application limitations of simulating some of the traditional experiences, mainly focusing on volumetric texturing tools and physical making in a research-based manner. The limitations of the existing application focus primarily on the basic model from the outer base shape with no volumetric texture, freeform modeling, or advanced sculpting tools. The challenges involved finding ways to develop a geometry texture generator and implement it in a new simulation process to bring the VP model into a more realistic form with developed experience in prototyping. We consider it essential, as shown in Fig. 1, to plan for user studies in VR that include the traditional pottery-making aspects in consideration with visual feedback, haptic feedback, gestural interfaces, 3D modeling, and collaborative design.

### Modeling for virtual pottery

We believe that VP making development is associated with developing the users’ experience. Our investigation contributes to finding the most reliable method, using available tools to simulate traditional pottery making from molding and physical characteristics through testing and analyzing existing VP systems. Furthermore, extending deformable shape modeling out of the basic cylindrical shape dimension coordinates available in modeling on VR throwing wheels.

### Interactive and collaborative virtual pottery

This research is about bringing pottery into the world of VR to explore new ways of experiencing the clay and pushing creativity. Indeed, there are many opportunities for VR to be used to enhance creativity and problem solving [3]. In a study, Yang et al. [4] showed that the participants in an immersive VR environment had higher quality creative products than those in the paper-and-pencil. In their recent paper, Lee et al. [5] provides new insights into the possibilities of using VR in collaborative exploration to promote creativity. Furthermore, Chang [6] highlighted the use of VR-based instruction positively affected the students’ Engineering Design results and Lin & Wang. [7] found that VR technology can effectively facilitate students’ creative self-efficacy and intrinsic motivation.

### Overall aim and objectives

This paper aims to develop the existing VP applications by adding the central missing factor of texturing, a novel volumetric sound texture for generating unique geometry patterns, including the development of VP modeling and user interaction making with a system refinement. Therefore, the paper is directed toward achieving the following objectives:

- **Modeling**: To develop and represent a volumetric sound resonance shape surface texture in real time.
- **User interaction**: To develop a multi-modal interaction that simulates physical-virtual to capture real-world deformable pottery making (addition/ subtraction) using tangible hand and finger transformations.
- **Making/ceramics**: The objective is to make sound texture along with physical making central to this process by emphasizing the material and the rapid prototyping, which influences the above two objectives.
- **System refinement:** specification of the system, system integration, and functional testing.

### Proposed virtual pottery

The authors of this paper feel that in order for a VR pottery system to afford an experience that effectively nurtures creativity, the usability of the VR technology needs to be considered. Creativity, on one side is about having the flexibility and fluidity within a system to create a novel pottery solution while usability, on the other hand, is about how effectively and efficiently it is to use the VR pottery system. Creativity and usability need to have a balanced relationship and if the VR pottery system is highly usable then this will have a knock-on effect on the creativity and pottery created.

### Organization of the paper

The paper is organized as follows as a blueprint guide map to understand how we form our approach on solid grounds and show the journey of our system with all of our contributions. Section 3 illustrates the related work to VR modeling in arts, design, edutainment, training, and VR usability. We discuss each of these below. Section 2 presents the overall aim of developing VP modeling and user’s experience gaining physical and visual knowledge with several contributions. Section 4 introduces some preliminary definitions. Our VP system software and tools present a simulation method of traditional pottery-making in Sect. 5. Section 6 examines a set of evaluation methods and describes our method for the VP modeling system, and conclusions are given in Sect.7.

## Contributions

This paper presents our aim of developing VP modeling and users experience gaining physical and visual knowledge with several contributions. Firstly, our method involves bringing the basic making methods and techniques of traditional pottery into one experience by comparing related VP research and potential evaluation methods. The experience starts with a basic cylindrical vessel on the VR wheel simulating the physical activity with haptic feedback for more realistic modeling of deformation and some limited trimming tools. Secondly, the user imports an X-change file to extend the process by free-form modeling using clay tools for clay texture representation and increasing the mesh count to extend the basic model out of the basic cylindrical dimensions. Thirdly, we apply our novel sound texture to the imported X-change file with a 3D printing tool to analyze and fix errors in the basic model and zero-faces in VP applications to be prepared for slicing. The major contributions of our work are as follows:

- VP system refinements by using integrated VP tools, Image-based texturing, and topology utilizing one X-change file, creating a closed bi-directional system modeling.
- Comparing related VP research and potential evaluation methods.
- Free-form modeling with mesh blending allows the user to create forms outside the basic coordinates.
- Apply novel sound volumetric texture pattern with a geometric generator of Chladni plate that uses mathematical functions.
- Locating topological errors in VP applications of seam-lines and zero faces.
- Advance traditional art with the existing VP modeling technique and experiment with the 3D fabrication process using experimental ceramic resin.
- VP systems refinements, developing the basic VP making toward freeform traditional experience simulation for practical collaborative and VR learning.

## Related work

Our work relates to the existing literature on VR modeling in arts, design, edutainment, training, and VR usability. We discuss each of these below.

### Creativity, VR, and usability

Graessler & Taplick ([8], p1) define creativity as a human ability that enables the development of innovative products. Creativity is about coming up with new and novel ideas, and it is about looking for alternatives, experimenting, pushing boundaries, expressing, and ultimately adding new value [8]. To be creative, Amabile et al. [9] highlight that a person must be surrounded by an environment that fosters and brings out creativity. The authors talk about intrinsic motivation as being conducive to creativity [10]. In fact, Stańko-Kaczmarek [11] found that intrinsically motivated art students experienced significantly higher levels of positive affect in the creative process and evaluated their performance significantly higher than extrinsically motivated students. In terms of pottery, several qualities in the clay can trigger the imagination and/or senses to reshape and create something new. Yet when it comes to creativity, it also can just be about the ability to perceive and experience clay and pottery in new ways.

###  VR modeling in art and design

VR modeling tools are a cutting edge technology with continuous development over the past decade. The exploration of creating 3D objects in a VR space with physical interaction provides novel opportunities for the fields of contemporary art and design to create an impact in diverse sectors, e.g., pottery, fine art architecture, interior design, landscape, and 3D design. The aim is to understand the spatial relationships within a 3D structure, to unleash the creativity of visual and physical interaction [12].

### Modelings techniques

*Free-form surface modeling:* Liverani et al. [13] presented a free-form surface modeling technique toward the integration of VR and CAD for free-form curve-based geometry in a 3D modeling system. The method is about a virtually integrated surface modeler (VISM) equipped with two advanced tools for 3D surface modeling, emphasizing VR visual feedback and improving complex surface modeling and creativity. The tools are for advanced skinning (PS) and curve-over-surface shaping (COSS), both implemented on a bimanual virtual environment (VE) to deform surfaces for true interactive modeling sessions.

*Subdivision surfaces:* Jia et al. [14] showed an algorithm for the subdivision of surfaces of revolution for most fundamental types of intersections—line/surface and surface intersections on cylindrical bounding hollowed shells. The method provides a robust technique for approximating complex generatrix curves using a series of biquadrics for virtual manufacturing, and assembly environments with components in the form of surface.

*Mesh blending:* Liu et al. [15] introduced the mesh blending method for smoothing, sharpening, and mesh editing. The technique showed the methodology of connecting various patches on triangle meshes with random connectivity. The significant characteristic of mesh blending is to move vertices of the blending region to a virtual blending surface by choosing an appropriate parameterization of vertices. The method gives the users the advantages of intuitively controlling the blending result using a different blending with cross-section curves that can be adjusted to flexibly design complex models. Finally, the resulting mesh has the same connectivity as the original mesh.

*Relief modeling:* Sourin [16] presented VR modeling technique of metal surface deformation plastically carving relief of virtual embossing based on the function representation of a metal plate and tools. His method simulates the process done interactively with virtual tools on a virtual sheet of metal with a photo-realistic appearance. The author developed a software tool that would be able to run on a typical PC to be able to do embossing virtually everywhere, even while flying in an airplane or sitting in the back seat of a car. Using the function representation for modeling provides any selected simulation precision, avoiding artifacts that are usual for polygonal representation.

*Volumetric sculpting:* Ferley et al. [17] presented a sculpting technique for rapid shape prototyping, using free-form tools. The method mimics local deformations, using tools like a stamp to make imprints on an existing shape. The approach also focuses on the rendering quality, exploiting lighting variations and environment textures that simulate good-quality highlights on the surface, enhancing the model understanding the spatial relationships. The implementation is based on GLUT and can run the application on Unix-based systems, such as Irix and Linux, and Windows.

*Structural enhancement:* Wang et al. [18] proposed a 3D solution for 3D prototyping in modeling of hollowing an object while maintaining the strength, reducing material cost, and meeting static stability of the model as it is one of the significant considerations for the users choice to minimize the usage of material. The method uses an algorithm system to enhance problematic structural regions, and adaptively hollow structural vital areas, while constraining by structural strength, static stability, and printability.

*Multi-level voxel modeling:* Biswas et al. [19] presented a 3D voxel printing technique for the discretization of a sphere in space, giving height to a set of mathematically precise, 3D printable physical voxels to the desired level of precision. The method was an efficient solution for voxelization and related discrete-geometric problems. Their work also attempts an accurate solution for sphere printing with an optimized length of the print head path and scalable resolution is determined by the voxel dimension.

### VP systems

The available systems are divided into two kinds: research and game-based. This section’s focus describes a variety of factors, showing the distinctive contribution in each one and the systems’ shortcomings. The systems are mainly aimed at novice users with a simulation system of traditional pottery, providing the VE with tools. The methods vary from VR kit, tools, and haptic feedback. This section aims to identify the most helpful tool in the thesis experiments and provide some knowledge of other tools.

*Research-based systems:* The research-based systems background gives more insights into the VP systems application with user usability evaluation, which has been tested but is mostly unavailable as an open-source for users and designed for specific VR kit tools. we present in the literature section the significant impact on our research:

Lin et al. [20] presented an immersive VP modeling system equipped with Leap Motion and HTC Vive. The VP system presented was involved in the actual pottery production process, using bare hands-free formation overcoming the limitations of physical circumstances. The basic shape used in the system is cylindrical with controlled deformation by Gaussian function to for basic pottery vessels. The Gaussian function forms a push/pull deformation on the actual mesh of the object, producing different heights of the curve’s peak on the cylindrical model by the user’s choice in various distributions. The approach gives the sense of reality and immersion of the knowledge mixed with motion capture and virtual reality technology. The usability test of the VP system was evaluated by a questionnaire immersion, completion, operative complication, and entertainment are carried out to improve the user’s experience.

POTEL is an engaging VR application that focuses on exploring the art of pottery modeling through VR [21]. The application is designed for novice users. It is an affordable system to model VP without holding any device to interact with environments is available as an open-source. Then the system uses an Oculus VR for visualizing the world, a Leap Motion for interaction with the environment, and an optional Arduino with mobile phone vibrators for haptic feedback. The modeling method relies on real-time deformation by extruding/compressing triangle vertices and interaction is achieved by pressing the user’s index finger. The users can bake their work, and by baking, it means saving information related to a 3D mesh into a texture file, then exporting it as an STL file for 3D printing after the experience.

Wowtao is a pottery manufacture system that expands VP creativity simulation [22]. The idea of the system is to design customized pottery virtually on tablets, computers, or mobile phones interactively in a short period. The application works with an integrated concept of customer product personalization, supporting unique designs in the pottery manufacture field. The project developers claim to simulate the entire physical pottery design process, such as modeling, painting, seals, and firing. The application provides the ability to have the designed results fabricated by real artists or a 3D printer. The approach of the application imposes some industrial constraints of geometry and decorations. The system focuses on novice users undertaking new skills of VP in the workflow efficiently within 10 minutes. The decorative process involves creating the 3D form in real-time with only visual image patterns with no volumetric texture deformation on the vessel. The Wowtao application can export files for 3D printing. Finally, the easy-to-use application for novice users was relatively similar to those experienced users from visual feedback trials.

PotteryGo is a VP training application system . Chiang et al. [23] highlight several ongoing challenges that still exist in virtual systems. VP brings several benefits in terms of experience in real-time rendering as the PotteryGo application offers deformable modeling to sensitivity to push/pull the virtual clay. The application focused on teaching fundamental knowledge and practical techniques in VR environments. The gesture analysis makes it possible to correct the learner’s actions by visual feedback. The application is aimed mainly for novice users, for extensive practice and skill gaining of traditional making simulation. The central aspect is creating a deformation by using the physical interaction through the Leap Motion sensor. The user’s hand gestures give input data information, making the pull/push effect create a mesh deformation. The visual feedback allows users to sync in the VR environment by three ways: auditive, depth cues, and haptic. The application can export STL files.

*VP Game-based systems:* The game-based systems background gives a different type of insight. The accessibility is either open-source or commercial systems available for purchase. The feedback users experience is direct on many websites, for example, Steam-powered. The game-based application can enhance the user experience, and the developers have semidirect feedback impacting the quality to be improved for a more immersive game experience. The liability of VP game-based can be approved or questioned by traditional pottery artists/makers.

Dojagi is a VR Korean pottery simulation game with a virtual potter’s workshop [24]. It is a game-based application for beginners and a spinning wheel simulation. The system of this VP application involves creative and training experience, and it is compatible with the HTC Vive/ Oculus Rift VR kits. The application has exceptional haptic feedback for a realistic indirect experience. The expertize simulation is made with the famous pottery artist Damggol Kim Jong-young to introduce a highly realistic simulation. The experience involves:

- Immersing hands in clay and developing skills making on VP wheel.
- Glazing.
- Sculpting, vernier calipers, trimming, measuring tools.
- Decorate/furniture of VR space studio.
- Change Visual VR tool.
- 50 pattern stamps.
- Water simulation for VP making.
- Throwing wheel (spinning).
- Export and archive files.
- Tutorials.

The applications of VR controllers can control the inner and outer sides of the VR object, creating a realistic deformation. The leading VR object uses an empty object limited to single coordinates. The vessel starts from a lump basic cylindrical clay model with a set of coordinates for the user choice. The haptic controller feedback shows a reliable simulation of the force of pull/push in and out of an object. The throwing/ spinning wheel has beneficial real-time feedback with the controllers. Adding the water concept significantly impacts the learning skills, an essential part of VP making. The VR sculpting tools help narrow down the shape vessel coordinate as an additional deformation method. On the other hand, the application has some errors creating zero faces and seam-line gaps affecting the 3D print file. Furthermore, the holes in the VR object are mainly in the primary object, and the gaps are increased while shaping the object.

VP systems include an automated mesh generator and an interactive model editor. The mesh can generate more a realistic clay in VR, and users can use the concept of deformable shape modeling to shape the virtual object based on real pottery making [2, 25]. The Wowtao VP system approach by Cai et al. [22] is about creating a topologically equivalent to double-layer of cylinders as a basic vessel, with an option to push or pull, without texture relief, mesh growth of a dynamic typology or sculpting tools. The most advanced approach was by Dojagi [24], a STEM application that creates more of the traditional experience of pottery making by using a VR kit and real space. The advantage of Dojagi’s system relies on the accuracy of push and pull motion on VR objects, with multiple tools to subtract as Voxel-based modeling.

### VR usability studies

Martinez et al. [26] presented performance tests such as task completion time, hand distance, pick errors, drop errors of user satisfaction gathered through the use of questionnaires and interviews. The interaction techniques were based on composite positioning and maneuvering concepts, designed, and evaluated in the context of a user-centered process. The authors found evidence that mixing natural with non-natural interaction in an immersive VE can confuse the users and make them face the system like a computer program instead of reusing their experience in the real world.

Zaidi et al. [27] propose a framework for usability evaluation of gamified VR and focus on how VR could be adopted for different purposes for different users and evidence that VR brings positive emotions. Wang, Cheng, and Luo [28] state that significant challenges are not well studied in the literature. For instance, these ideas still need to be expanded to a gamified VP application.

Pietrowicz et al. [29] explained that virtual worlds are 3D simulations of real or imagined worlds, extending reality by integrating experience between the physical and virtual worlds, implementing even more possibilities. The human interaction in VR is more expressive, influential, and dynamic human control paradigm. They investigate high-performance interfaces modeled after the techniques of musicians and other performing artists. The developments employ automated learning skills and data mining methods to extract features from multiple data streams such as: audio, video, and motion capture.

Casu et al. [30] showed that recent developments in user hardware lowers the cost limitation for adopting immersive VR solutions. They introduced RiftArt, a VR tool for supporting the teaching and studying of Art History. RiftArt configures virtual museum rooms with artwork models inside and enhances them with multimodal annotation, supporting the teachers throughout the lessons and the students during rehearsal. They also demonstrate how VR increases the motivation of high-school students’motivation to study Art History, and they provide a deep analysis of the factors that contribute to the results.

De Klerk et al. [31] explained how VR could assist architects at the early stages of creativity and design. They built and explored maquettes at different scales in early design stages. They developed a VR environment where untethered, easy-to-operate peripherals support user interactions, using VR headsets to provide virtual immersion and simplified geometric shapes to model voxel-based maquettes. They also show how usability studies with laypeople suggest that the proposed system is easier to use and more effective than the current CAD software to create simplified models rapidly. The results play a visible role in supporting the creative process, allowing architects to become both builders and explorers of spatial constructs.

Finally, Lteif et al. [32] presented an observational study to integrate VR technology with art. The study was conducted by replicating an actual museum by a Swiss-Lebanese artist and having the users experience it in an immersive VE. The study collected data to monitor the user experience and its effects on the emotional and psychological state. The collected data revealed the developments and enhancements, elevating the user experience and possibly improving and enhancing it with more advanced technologies.

### VR in STEM edutainment and training

Gao et al. [33] propose an interactive installation for VP, which uses motion sensing to understand ceramics art better. Arango and Neira [21] present a VP system that uses affordable (low cost) technology, allowing novices to understand the experience of traditional making as a game, gaining new skills, not as a research-based experience. However, in both systems, the physical feeling of pottery-making is absent due to the use of mid-air gestures. Smith et al. [34] describes the uses of VR game environments in a multi-disciplinary context, enhancing discipline-specific skills of graphical realism in a simulated environment, and the ability of VR environment to improve the learning experience and collaboration across several disciplines, e.g., computing, construction, management, nursing, and midwifery.

Villagrasa et al. [35] presented a method of gamification integrated with VR, enhancing the learning methodology for architecture classes. Their overall aim is to increase student/user engagement in a classroom through creative technologies based on game mechanics. They focused on this type of recognition as an essential element when considering gamification. They used Unity, Sketchfab, and Oculus Rift systems to develop high-quality and realistic worlds. This process generates a highly interactive classroom and promotes the outcome to remain more detailed and at a more satisfactory level.

Guan et al. [36] showed the effects of a VR-based pottery making approach on junior high school students, examining creative ability, learning engagement, and opportunities to practice new skills. This approach examines students’ ability to receive VR instruction. It is a meaningful approach to construct observation of physical/visual interaction and reflection on the process for students in a pottery making class, promoting their learning experience and performance using VR tools. The study also showed an analysis of group study of VR-based approach that had a higher cognitive engagement than other groups of those in the control groups with paper-and-pencil and clay. This approach of VR modeling showed more creativity in VR space and interaction with VR objects.

### Improving virtual prototyping

Müller et al. [37] investigate virtual prototyping guidelines of interaction and visualization techniques to overcome current limitations, evaluating the user interface concepts in integrating VR in a participatory design process, and introduce agricultural machinery and the automotive industry as application scenarios for VR prototyping, as well as, emphasizing how the current technological advancement in virtual prototypes are affordable, quickly realized, and easy to modify.

## Preliminaries

This paper seeks to address the appropriate evaluation methods, evaluating our novel VP system’s usability of a multi-disciplinary engagement approach, e.g., VR 3D modeling, games [38]. The motivation of our system is to encourage using deformable shape modeling in VR with newly developed insights that can help many fields [39], e.g., digital twin, edutainment curricula, and training skills. VP modeling, and fabrication have several opportunities, challenges, and desirable properties:

- **VP usability evaluation methods:** VP game applications are still in the development stages, filling the knowledge gap to be considered a reliable representation tool of traditional making. The advancement methodology of VP application is trying to improve user skills, technique, and fabrication for physical/visual interaction modeling. Today’s innovation of cutting edge enhanced technology, assets on developing a more reliable sustainable usability evaluation in research.
- **Labor and compute intensive:** Many sophisticated methods for 3D deformable shape modeling are available to produce realistic outputs, but they are still considered as labor-intensive, expensive, and time-consuming.
- **Creative technology integration for VP:** Nowadays, innovative technologies are more involved in the art field of developing human intelligence, performance, and fabrication. VP is one of the data physicalization methods, demonstrating real-time active deformable shape modeling, and it is currently a rendered technique of representing an actual data physicalization. There is a need to create a new form of integrating applications and tools to perform an improved experience and VP model.
- **VP analysis:** VP is a method that focuses on users physical/visual interaction to create complex VP objects, using digital clay representing the pottery making in real-time. Thus, developers of VP applications still lack some knowledge in some of the tasks performed by professional makers, for developing the virtual clay object to represent the whole traditional pottery making.
- **User manual and teacher/trainer for VP:** VP applications are acknowledged as a game of one player to experience the basic making techniques with some helpful tools. There are no communication methods of teacher/trainer to guide users development other than simple tutorials or figures to cope with on-the-go simple instructions.

In summary, VR in pottery boosts creativity and functions as a problem-solving method. This is realized by experiencing the VP system we present in our work. The concept of VR modeling creativity is about the unique path of creating complex objects virtually. The VP making actively nurtures creativity and focuses on the usability of VR technology, and it must be considered a method of improving human ability. The remaining sections are organized as follows. The following section presents related work. Then we look at the usability of VR systems in the context of VP. Finally, we present the design aspects of our system, and its experimental realization.

## Our VP system software and tools

### Design and implementation

**Fig. 2:**
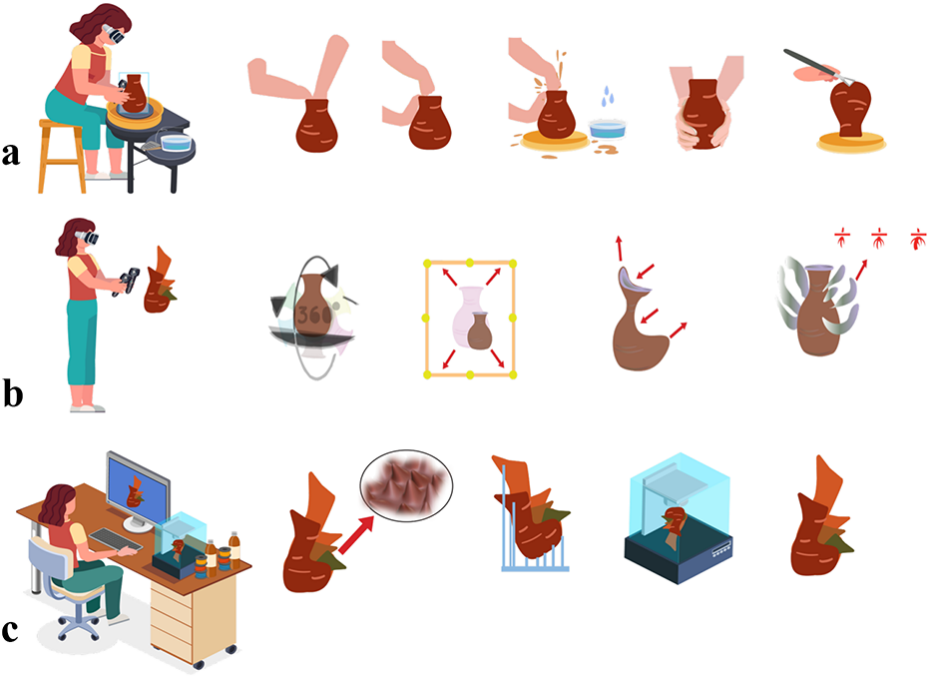
Overview of our VP system

Our proposed system is an interactive virtual physical 3D modeling for a multi-disciplinary approach, e.g., Edutainment and games. [40]. Figure 2 shows a schematic overview of how the user can operate the VP system. The system uses integrated methods of operation starting from modeling toward fabrication, showing the difference of interactive physical activity and visual feedback reflected on VP modeling: Fig. 2a shows the users’ first step in a seated position, modeling on a virtual throwing wheel to create the primary VP object, showing the basics modeling technique with water simulation and trimming tools.

Figure 2b shows the second VR modeling step in a VR sculpting application, extending the VR object in a standing or seated position. This step is basically for exploring modeling interaction and sculpting tools within the selected area boundaries in the lab/room, Fig. 2c is the phase where the user uses a 3D modeling application to add our novel texture with a 3D printing tool to fix any errors, preparing for the next slicing phase as preparation for fabrication step for the user to prototype the X-change file using a 3D printer.

**Fig. 3:**
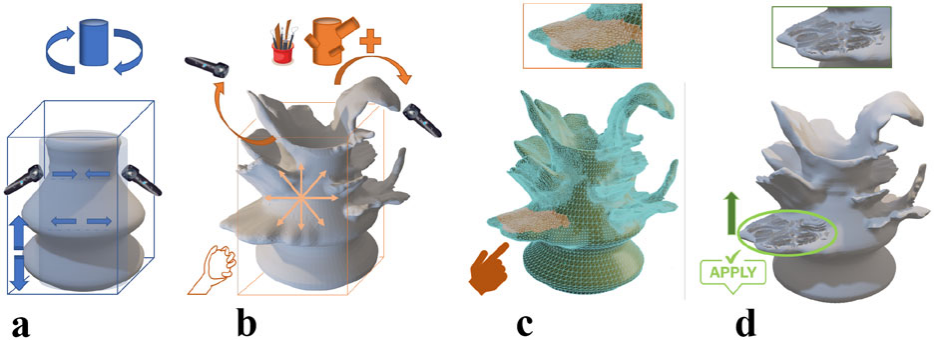
Novel VP deformable shape modeling workflow

In Fig. 3, we show how our method of modeling works and how we extend the existing VP modeling application. In Fig. 3a, we see the basic cylindrical model approach of VP modeling in fixed dimension coordinates, deforming the VR clay by push, pull hollowing the object to create a vessel. In Fig. 3b, we present the start of our novel part extending the VR object, using an X-change file toward freeform modeling, adding the clay material properties. This approach uses the user’s physical interactive modeling data from the controller’s haptic feedback as a unique texture and extends the mesh count using the pull feature to reform the VP object, allowing the user to simulate traditional pottery making. In Fig. 3c, d we show the textured relief or carving as it is one of the critical aspects of pottery making, and how the high data of texturing would affect VP application.

**Fig. 4:**
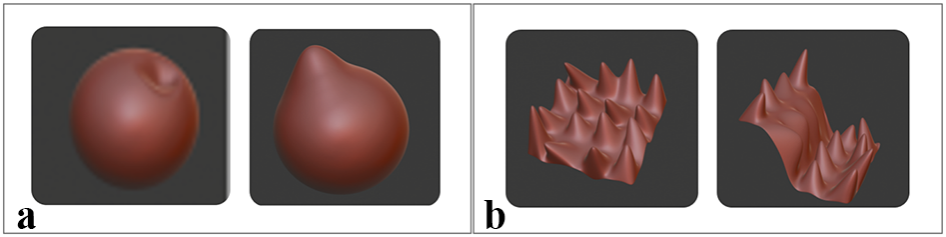
Texture deformation properties

We use the term X-change to represent a suitable file format. The OBJ and STL formats are used in our experiments and tools to exchange files between the systems. We tried to maneuver and use the same X-change file to apply texture on the shell surface of the VP model. Our technique of texture application creates not only a relief but also the texture interacts with mesh shape, and the texture is deformed not only by pushing as shown in Fig. 4a and pulling, but also the stretch shown in Fig. 4b properties to be reformed with the existing mesh on the shell surface, which the user could not get the same outcome when using clay.

The mesh blending method here works in two different ways. The first method is by using Dynamic topology in VR applications. The second method works by integrating a single standard texture map on the 3D mesh, producing a smooth join between different map images on the surface sections based on texture blending. The accuracy of sound resonance texture shows a more promising output than the procedural noise methods, corresponding to the mesh’s triangles.

We used the mesh blending approach in the Blender tool by subdividing the selected vertex group, using less weight than the whole object (0.2–1). Then we displaced the selected mesh with a selection of texture maps. The subdivision levels in the 3D Viewport or the final render increase the levels of subdivisions, resulting in additional vertices. This method automatically blends the function, allowing the user to smoothly control the blending result by a user-specified blending radius and provides the desired mode of designing complex textures. The mesh blending method can be widely used in smoothing, sharpening, and mesh editing, using data from texture maps.

**Fig. 5:**
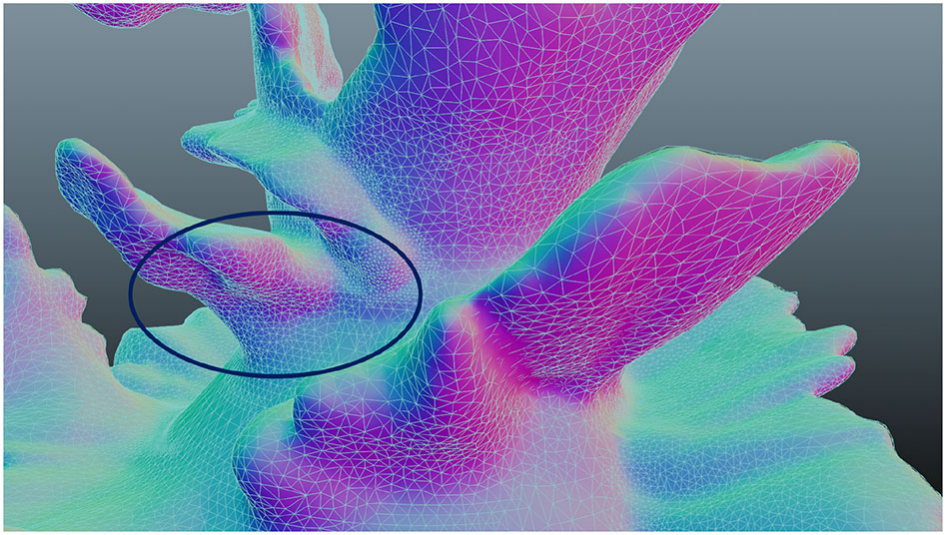
Mesh blending

After modeling the VR object made with a VP throwing wheel, we extended the model with another step, using a sculpting application, as shown in Fig. 3b, which works as a tool that applies mesh blending as shown in Fig. 5 technique, adding the mesh count on the original basic cylindrical shape.

1. Throwing wheel is a VR system that is used to create the base object.
2. The base object is also deformed sideways on the throwing wheel.
3. In the next step, a sculpting application is used to make more detailed deformations. The meshes can also be constructed (added/subtracted) in the sculpting tool.

The next stage is to blend the Chladini texture mesh in the Blender tool.

This approach allows bringing the user creativity of smooth blending as if attaching new parts of clay on the basic model.

**Fig. 6:**
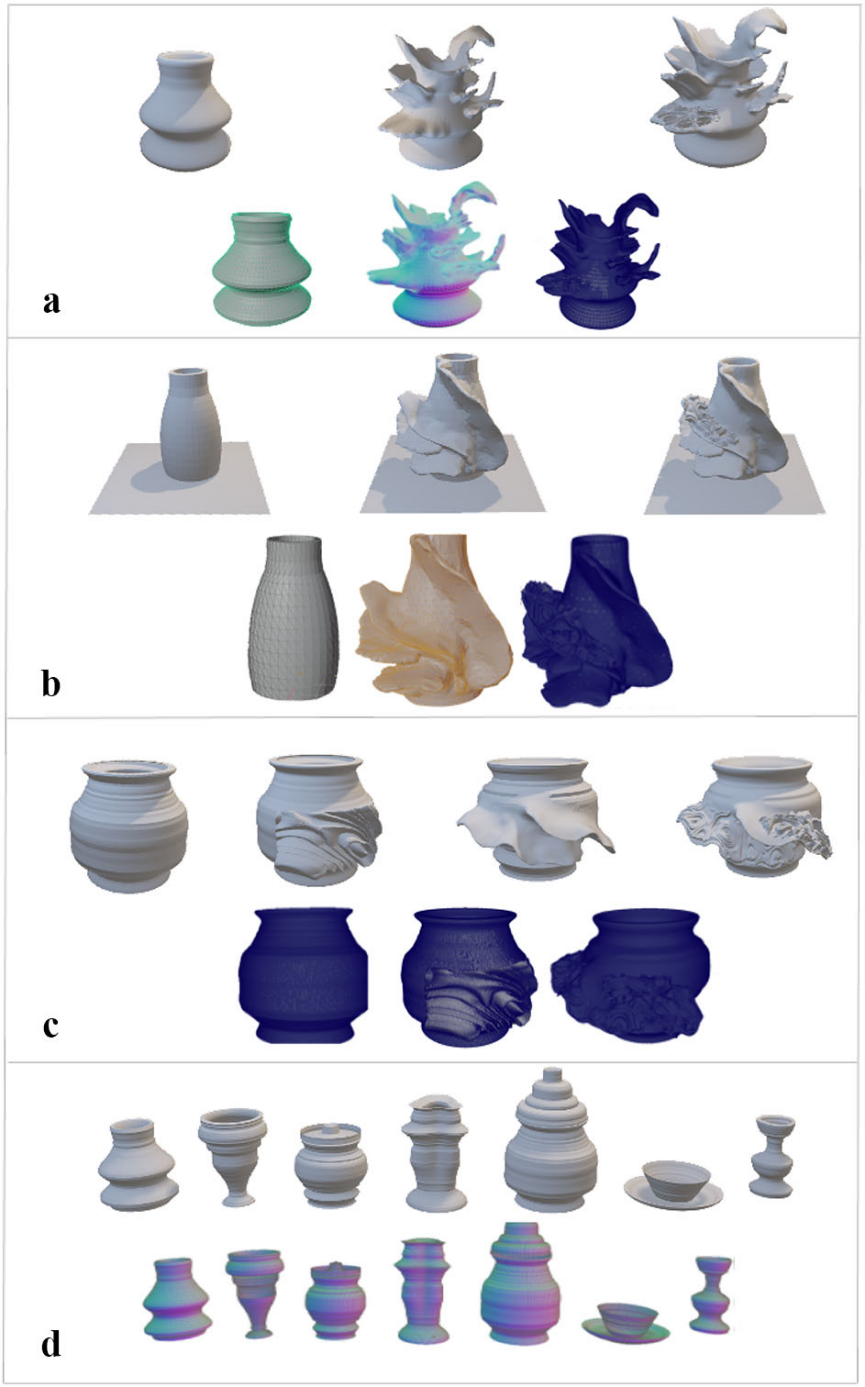
VP modeling outputs

The modeling outputs of our VP systems are in 3D view visualization and wire frame shown in Fig. 6, Fig. 6a shows a complete process outputs of our approach using VP throwing wheel with VR sculpting tools and 3D texture application, Fig. 6b and 6c presents how to use an X-change file and how to add clay deformation with texture application, Fig. 6d presents a range of pot modeled using our system, showing the difference between adding 3D texture direct on the VP model from the left and the right adding texture on the extended layers of original VP object.

### VP use case and usability design

**Fig. 7:**
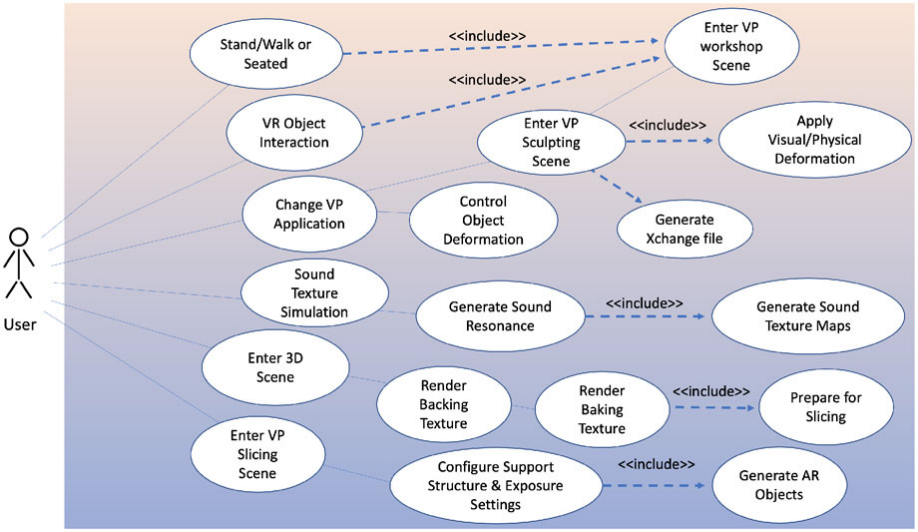
Use case diagram

**Fig. 8:**
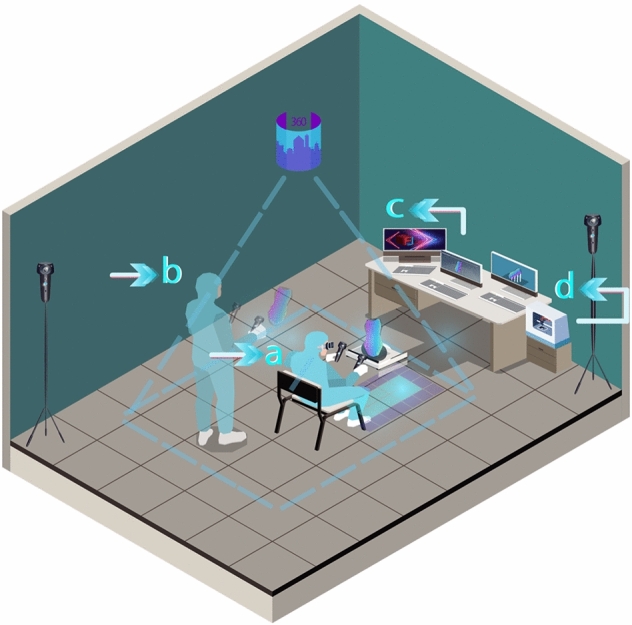
Design and usability overview of our novel VP system

The system is chosen to extend ceramic artists’ creativity of making, scaling up the boundaries of creative making, and developing a physical-virtual experience that captures real-world deformable pottery making (addition/subtraction) using tangible hand, and finger transformation. The next step is to capture the requirements from the schematic to define the use cases/ actions that a user would perform. This helps in the systematic design of the VP system from a user’s perspective. The use case diagram is shown in Fig. 7. The usability design and room setup is shown in Fig. 8, and it reflects how the user’s positions are importantly reflecting on the modeling, Fig. 8a shows the seated position using VR wheel. Presenting the importance of the position with haptic controller feedback brings more realism to VP modeling. Figure 8b is the position of examining the output model in 360 views from the last step and how to reform the VP model within marked space. Figure 8c is a computed-based phase to apply 3D texture with fixing model errors. Figure 8d is the final step of 3D printing.

## Evaluation

In this section, we present a separate related work that are evaluated on multiple criteria, that are set from pottery makers perspective as seen in Tables 1, 2, 5, 4, 5 and 6 from modeling, users experience, outputs, usability, and then comparing it with our VP system, presenting potential evaluation methods. Our evaluation methods highlight what each VP system provides to simulate a better traditional pottery-making modeling toward fabricating experience. Also, our VP system guidelines are for the user’s choice of the tool and methodology to show how to extend deformable shape molding through our novel texture, technique, and user modeling experience. In the following section, we firstly present our VP system. We then show comparative VP systems analysis with the outcome of gaming using our system reflected in Digital Twin. Next, explore some related user studies, visual feedback, VR test-bed evaluation, and finally, a demonstration of comparative analysis.

**Table 1:** VP system comparison and users experience criteria

VP systems	Wowtao (Training)	PotteryGo (Training)	POTEL (Training)	Our VP system (Sound Modeling System) Game play / Training
User interface	Touch/Tablet	Gestures/PC	Gestures	Tracked motion controllers
Workflow	Includes: Modeling, painting/image pattern, bottom design, firing and final visual image.	Includes: Learning, interactive training, making vessel, surface painting/ free creation and share/teaching.	Creating a vessel in real-time pull/push, brush deformation, bake to STL.	Create vessel in VR space, extend VR object growth, adding sound shape surface texture, experimental slicing, prototype, and link physical object with AR
System setup	$\bullet$ Tablet/Smartphone.	$\bullet$ PC -Head set – Room Scale/seated Leap motion hand.	$\bullet$ Tablet PC -Head set – Room scale/seated leap motion hand.	$\bullet$ VR kit/ VR space
	$\bullet$ Fixed Environment.	$\bullet$ Fixed environment.	$\bullet$ Fixed environment.	$\bullet$ Dojagi & shapeLab application features:
				$\bullet$ Room-scale/seated & standing.
				$\bullet$ VR wheel/ paddle speed controller/ kiln.
				$\bullet$ VR tools/ graver, measurement tool/ vernier caliper and Brush.
				$\bullet$ Design own workplace and appliances.
Training module	$\checkmark$	$\checkmark$	$\checkmark$	$\checkmark$
User study/Evaluation of visual feedback	Easy-to-use even for novice users were rather similar to those from the experienced users from visual feedback trials.	Strong effect of visual feedback on participants to learn pottery-making skills.	The visual feedback allows user to sync in VR environment by three ways Auditive, depth cues and haptic.	Future research step: the visual feedback may have effect on extending pottery making of creating a complex VR object and developing it in a multiple application with rapid prototyping.

### Our VP system

**Fig. 9:**
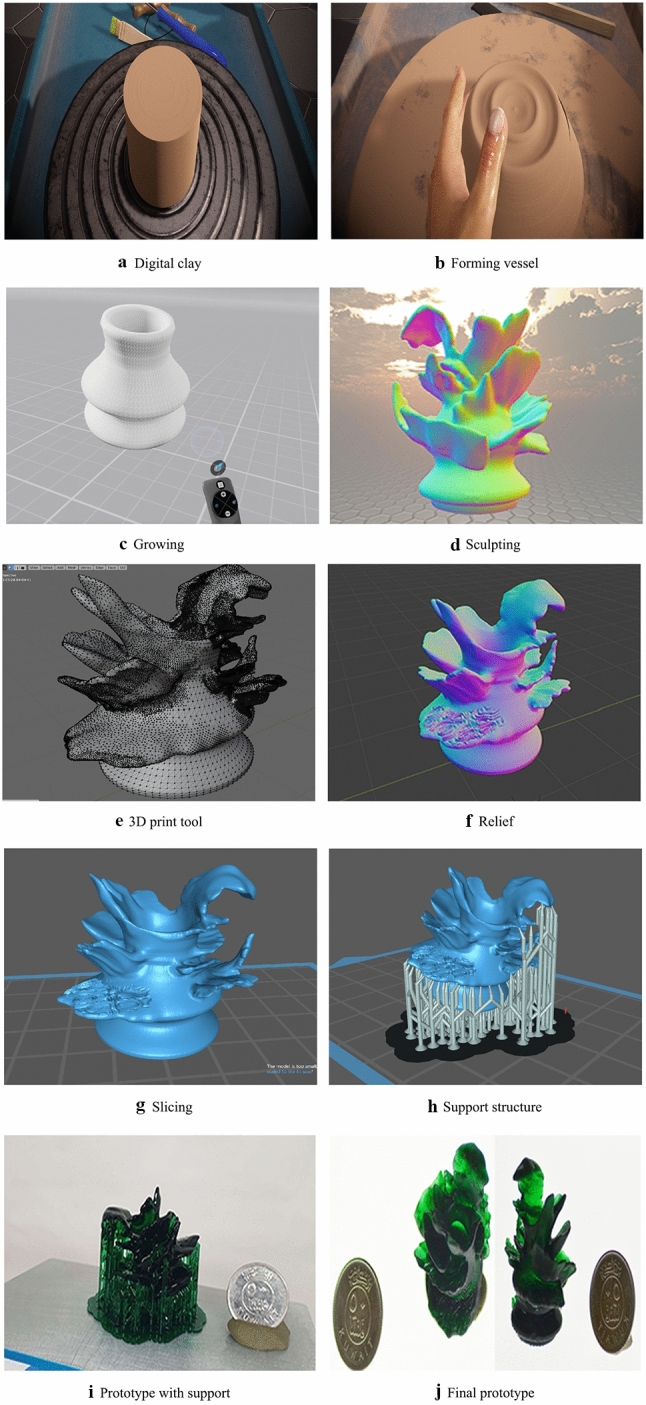
Forming and prototyping a virtual clay vessel

As shown in Fig. 9a, b, c, d, e, f, g, h, i, j, the proposed system integrates a chain of VR, 3D, and fabricating applications with new techniques to simulate as much as the user would experience in the real traditional making using our VP system. The overview of our system usability is described in Tables 1 and 2 explains the steps from learning to fabricating how the user would assess the development, and how we evaluate the user’s experience.

The evaluation presented in the paper includes (i) visual comparison of relief synthesis compared to other research presented in the literature; (ii) the interaction models proposed and implemented in this research compared to interaction models used by other researchers; (iii) user studies target creation of simple shape deformation and addition of 2D textures. However, our prototype uses multiple systems during the modeling stage, which requires sufficient knowledge in the use of the systems to model the shape with sound deformation. Dojagi and ShapeLab are the appropriate applications for our project to support VP. More so, enhancing the ability of basic and intermediate users to analyze VR models errors and extending surface deformation of image-based modeling through using Blender software and experimental slicer settings. We present a comparison of VP application in Table 3 to explain how our systems is compatible with the Dojagi VP application due to the viable elements within the application and the ability to export printable files shown in Table 4.

### Comparative VP systems analysis

**Fig. 10:**
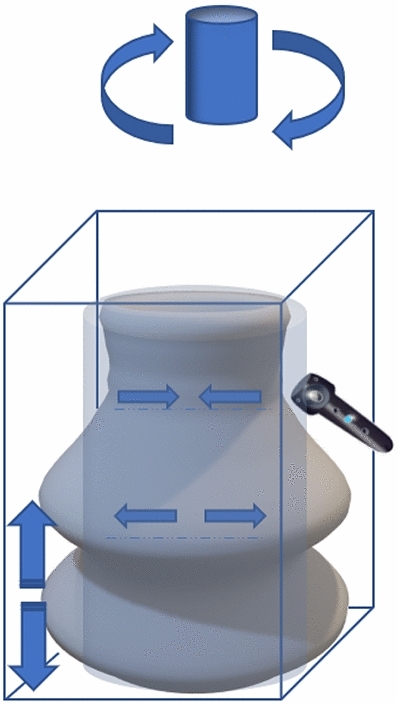
VP basic cylindrical shape

This section demonstrates how our system is different from some couple of system shown in the form of a table. A systematic approach for analyzing and utilizing data to examine the effectiveness and efficiency of VP systems. Table 5 brings the essential elements of a VP system to convey the most satisfactory of each system showing why we chose Dojagi as a basic VP application for VR throwing and how user interaction of interactive modeling in and out of the object as shown in Figs. 2a, 9a, b, 3 and 6. The most important realization in basic modeling aspects is that all of the systems works on a basic cylindrical shape with a single coordinate point with no additional geometry as shown in Fig. 10.

**Table 2:** VR prototype demonstration: comparison with related work

Comparison	Shamsuzzoha et al.	Quinn et al.	Dashti et al.
Framework	Five layers for database, product data model and maintenance, VR system monitoring and evaluation	VR structure modeler, reaction simulator, VR visualizer	VR for modeling, texture synthesis, blending shapes, slicing and prototyping
Technology and tools	Unreal Engine 4, HTC Vive, HTC Vive headset, HTC Vive controllers, HTC Vive base station, Vive tracker, etc., (VIVE, 2018).	Rhino/ Grasshopper, dynamic relaxation solver Kangaroo, rendering and design of unity; SteamVR; mixed reality Hololens	HTC Vive kit, steam apps, chladni plate, materialize, blender and 3D printers
Use cases	Engine maintenance	Structural modeling; physics simulation, VR rendering; data transfer	Shape generation; sound integration; visualization, slicing
Demonstration	The application of VR in industrial operations and maintenance	VR for structural modeling and interaction	VR for VP
Physical prototyping	No	No	Yes, Clay

**Table 3:** VP systems modeling comparison criteria

VP modeling systems	Wowtao	Pottery-Go	POTEL	Our VP system (Sound Modeling System)
Deformation process / Types of gestures	$\bullet$ Real-time rendering	$\bullet$ Real-time rendering	$\bullet$ Real-time rendering	$\bullet$ Real-time rendering
	$\bullet$ Push/ Pull	$\bullet$ Raise up & top dig	$\bullet$ Raise up & top dig	$\bullet$ Raise up & top dig
				$\bullet$ Dynamic topology/ Growth
				$\bullet$ Imaged based-modeling relief
				$\bullet$ Free form modeling
				$\bullet$ 3D print tool / Fix Non-Manifold mesh
				$\bullet$ Table 3 VP systems prototyping criteria
2D Relief modeling / Surface detail	Image pattern (visual)	–	–	Sound resonance fractal images from (Chladni Plate)
3D Texture / Surface detail	–	–	–	Sound resonance fractal images from (Chladni Plate) + displacement and bump mapping, controlled by normal-map colors for height and depth

**Table 4:** VP systems prototyping comparison criteria

VP systems	Wowtao	Pottery-Go	POTEL	Our VP system (Sound Modeling System)
Export	–	$\checkmark$	X-change STL	X-change STL/ Obj
Slicing/ Support structure	Default FDM slicer	–	Default FDM slicer	Experimental FDM & DLP slicers
Relief prototyping	–	–	–	$\checkmark$

**Table 5:** Comparison of VP systems

Systems elements	[2] Realpot: an immersive virtual pottery system with handheld haptic devices	[22] Wowtao: a personalized pottery-making system	[24] UTPLUS Interactive VR STEAM application. Dojagi	[25] Turn: a virtual pottery by real spinning wheel	[41] PotteryGo: a virtual pottery making training system	Our VP system
Game/ Research based	Research	Research	Game	Research	Game & Research	Game & Research
System type	Interactive deformation system	Interactive deformation system	Interactive deformation system	Interactive deformation system	Interactive deformation system	Integrated interactive deformation system
User interface	VR UI	Smart-phone (tablet computer)	VR UI	Computer screen	Finger gestures on a tablet PC	$\bullet$ VR UI $\bullet$ PC
Modeling form	Basic cylindrical shape	Basic cylindrical shape	Basic cylindrical shape	Basic cylindrical shape	Basic cylindrical shape	Cylindrical + free form deformation
Object coordinates	Single coordinate point with no additional geometry	Single coordinate point with no additional geometry	Single coordinate point with no additional geometry	Single coordinate point with no additional geometry	single coordinate point with no additional geometry	$\bullet$ Free form deformation $\bullet$ Adding mesh count on basic cylindrical shape
Deformation	Basic deformation	Basic deformation	Basic deformation	Basic deformation	Basic deformation	Basic deformation + free-form deformation
Surface texture	–	–	–	–	–	Volumetric texture
Mesh count	Basic	Basic	Basic	Basic	Basic	Increase mesh count
Modeling tools	Basic	Basic	Advanced	Basic	Basic	Advanced (Integrated VR/3D tools)
Export	OBJ	X-change file	X-change file	–	–	X-change file
3D print output	–	Yes	Yes	–	–	Yes
User interaction	Basic VR simulation interaction	Touch-screen based interactions	Advanced pottery making simulation	Kinect sensor based interaction	Leap motion sensor interaction	Advanced pottery making simulation

**Table 6:** Skills table

Skills	Basic	Intermediate	Advanced
e-Learning	Short e-tutorials	e-course learning	Advanced self-learning and model development.
Gaming	Ability to understand and follow technical soft skills. Create on seat	Ability to recreate objects Set/ Walk in VR space	Ability to master control VR kit tools. Function under pressure; Create new models; Set/ Walk in VR space
VR Pottery/ Making	Under-standing basic pottery making; hand-building	Intermediate: throwing; hand-building; Sculpting.	Advanced deformation: Mantaflow; surface relief; Design new deformation algorithms. Mantaflow
Creativity	Minimal object deformation on VR object	Creating objects using: Mixed method of VR and 3D modeling	Developing /designing VR application for art output Combination of VR and physical object (augmented-reality)
3D Printing	Use normal settings	Analyzing object errors using 3D print tool (changing slicing settings)	Develop new slicing setting Analyzing errors in the designs in VR/3D (changing settings)

### Evaluating through narrative and gaming using digital twins

We have presented a set of evaluation methods and described our method for VP. Our system incorporates all the stages of the pottery fabrication cycle relying on digital tools and ends up with a 3D printed prototype. As Pitkänen, Iwata and Laru ([42], p. 1) state: “planning and facilitating digital fabrication activities, where students engage in creating tangible artifacts with digital technology, requires knowledge on both technology and pedagogy.” In fact, there are several systems that are aimed at VP fabrication, and evaluations should be part of the educational process. However, in many proposals, evaluation were limited to the opinion about a visual outcome, and immersion is limited to what can be done with visual feedback. Some of these systems are discussed next.

Arango and Neira [21] present a game where clay can be deformed using mesh extrusion, and compression , and the player can print in 3D the outcome. They [21] presented a sequence of steps which include sitting on a chair; pulling and pushing clay; modeling the clay by moving the palms and printing performed using head-mounted displays and haptic devices. However, the experience can be further improved to make it more accurate and immersive. To inform a fabrication system, Devendorf and Rosner [43] use contemporary performance art (diverse live presentations by artists). They ([43], p.562) claim that “histories, public space, time, environment, audiences, and gestures can be meaningfully enacted in fabrication activities.” Gao et al. [33] present a VP interactive installation that uses in-air hand interaction and state that the users can have the chance to express themselves as artists and that the development can be applied to arts education, entertainment, and exhibits. However, because of the technology used (Kinect), they missed the chance to produce more immersion through haptic sensations.

Chiang et al. [41] explain a serious game which assists beginners that learning the gestures used in pottery-making. Here, traditional pottery steps are respected, and authors conduct a set of experiments to evaluate the system, demonstrating the efficacy of the application in “helping novices learn the manual skills of pottery making as well as the applicability of these skills when applied in a pottery studio ([41], p.87).” However, the Leap-Motion used technology impedes haptic sensations from the user, and fine details cannot be managed. Li et al. [44] develop a VP gesture recognition library for teaching. Again, the system focuses on visual feedback for immersion. Finally, Liu, Zhang and Wan [45] describe a system to unroll 2D paintings on pottery surfaces, a method which in the future could be used to analyze pottery pictorially.

On the other hand, Digital Twins [46] are computerized clones of physical assets used for in-depth analysis and are the core technology for smart manufacturing [47]. Additionally, Gamification [48] reuses game design and elements in other contexts, such as virtual learning environments, where the purpose is to promote a better user experience by improving motivation and engagement. Indeed, VR games can be used for manufacturing education. A novel idea could allow us to integrate our VP application using gamification and digital twins’ technology to create a serious game that could allow users (art students, tourists, museum visitors, pottery factory visitors, pottery artists, among others) to understand and refine their pottery fabrication process. First, the user should be involved in a narrative (for example, becoming a pottery artist from an ancient civilization) in an approach similar to that proposed by Navarro-Newball et al. [49], which allows the recreation of life-like practices enacting a character immersed in a situation, to understand cultural and scientific practices. Additionally, we could think about including a digital twin in an approach similar to that proposed by Kolivand et al. [50]. Here, the idea would be connecting a pottery apprentice with an instructor. Both could be connected through a digital twin where the instructor could observe, correct, and decide to learn activities and evaluate not only the outcome of the fabrication process, while the apprentice could make questions that could interactively affect the instructor’s production.

Moreover, we could further explore interaction with a real pottery artist. With gamification, each step of the VP process could be associated with a game level where the user could gain some points. Following the practitioner’s progress would only require checking the score achieved by the learner at each level. The score could be obtained, not only using visual feedback, but also from data gained from each step and from the digital twin. Overall, this would allow a better evaluation of the VP process, including objective data and subjective opinions about the process.

### User studies

**Fig. 11:**
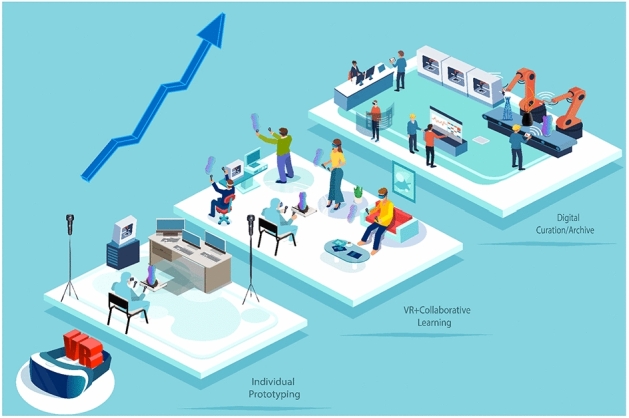
VR and immersive experience in different environments

We explore usability studies to enhance our VP systems, improving the user’s development of 3D deformable modeling for compatibility. Exploring the study methods will set the foundation of our future tests to support assessing the evaluation phase. The usability tests approach focuses on the criteria of a multi-disciplinary approach, integrating VR application for a novel outcome of graphics modeling and rapid prototyping. In the meantime, due to Covid-19 health restriction relying on research related to the field, we could not proceed with the full suite of user tests. We created an alternative plan where we have set a skill Table 6 for our usability studies to evaluate the users’ performance while experiencing our VP system. The system aims to assist novices, practitioners, and researchers in improving VP system concepts and applications, as shown in Fig. 11 starting from (a) individual prototyping, (b) toward collaborative learning in a safe place, and (c) digital curation/archive for fabrication.

### Visual feedback

Fulvio et al. [51] discuss how VR creates a bridge connection of 3D motion between the user development experience, VE and the consequences of their actions, improving performance while maintaining experimental control. Our VP system connects to this concept as the users develop their performance, using multiple applications to create a complex organic model from the visual feedback, improving rapid prototyping with physical/virtual modeling.

#### Gestural interfaces

Norman et al. [52] proposed the idea of the importance of the usability of *G*estural Interfaces. The true advantage of the Graphical User Interface (GUI), must focus on controlling the system so the users can understand the connection between the actions and the results of the outcomes.

#### Usability factors of 3D modeling in VR

Huang et al. [53] present the study to examine learning usability factors that affect the performance of 3D modeling in VR and investigate the ease of use of VR environment, using the System Usability Scale (SUS) for 3D modeling industrial design in VR. The study by Rieuf et al. [54, 55] suggests that VR technology can help industrial designers develop product design concepts. The study shows how the users believe that richer and more convenient functions could improve the usability of 3D modeling in VR. Also, the aspect of VR assists users to define the state of the virtual object sizes, distances and spatial understanding in VR.

#### Collaborative design for VR usability

Mahdjoub et al. [56] present the collaborative design for usability approach of modifying the product or the workplace virtual prototype with VR tools. VR tools are integrated based on a Multi-Agent System for knowledge management in mechanical design projects. The study focuses on developing the knowledge of engineering systems integrated into a PLM - Product Lifecycle Management – environment linked with VR tools, analyzing aspects of the virtual prototype for manufacturing and maintenance knowledge management approach, improving the collaborative design in industrial areas.

### VR test-bed evaluation

Bowman et al. [57] revealed how test-bed evaluation is an improvement for usability testing, suggesting how VE are becoming complex and how the designers are in need of guidance in choosing 3D interaction techniques. Also, how it is an effective and useful method for the assessment of interaction techniques for VE-focusing on the tasks and interaction requirements for applications. In terms of the performance technique, it requires high levels of spatial awareness and information gathering to improve the experience.

In summary, the evaluation guidelines highlighted in this section have provided a strong foundation in designing the interfaces for our PotteryVR system. The following section demonstrates key features of the system.

### Demonstration – a comparative analysis

The demonstrations of two comparable systems with our VP system are presented , and key aspects are captured in Table 2. In the StructVR system [58], the demonstration of the prototype provides immediate visual feedback of virtual physical interaction of 3D structures. The StructVR demonstrates the proof-of-concept prototype and discusses its technical realization. The phenomena visualized are the shape modeling of the 3D structures and the engineering properties of the structures, including deformation, forces and reactions. Even though different software systems could have been used to build the prototype, the prototype has been realized using Kangaroo Dynamic Relaxation (DR) solver due to the precision, speed and customizability of the structural deformations and also, due to the flexibility of the Rhino/Grasshopper software environment. The choice and justification of the tools are (i) geometric power and scripting versatility of Rhino/Grasshopper; (ii) the mechanical precision and speed of Kangaroo’s DR solver; (iii) the high-fidelity VR performance and design suite of Unity and (iv) Vive and steam for the user interaction and head mounted Display Graphics. The data exchange in the demonstration prototype uses mesh data including vertices and metadata including points, lines and extrusions that are transferred/exchanged between the systems. To compute rapid reactions, a DR solver within Kangaroo is used as opposed to a full 6-Degrees of Freedom implicit integration solver. In the Digital factory by Shamsuzzoha et al. [59] present the prototype for VR environment in a digital factory for industrial training and maintenance. The specific application demonstrated is for power plant operations and maintenance. The prototype demonstration had several elements: (i) the framework for the VR maintenance environment; (ii) Virtual prototyping to enhance VR maintenance environment, (iii) creation of the VR operational environment, tools, and techniques; (iv) requirements and use cases, (v) demonstration of the VR case study, and (vi) empirical and managerial implications. Each layer has its characteristics and significance and could adapt by changing individual layers.

- In the framework for the VR maintenance environment, the system is conceived as a vertical five-layer framework, where all the core data sits at the bottom. The data included CAD data of the shape models, maintenance data, and other relevant data. The layer above the basic data is the Data Management element. The framework’s middle layer is the maintenance task, scheduling, knowledge and resource management. The VR system layer is conceived the top of the management layer and the monitoring and evaluation sit on the top.
- For building the virtual prototype, the digital product model are utilized. Essential simulation and maintenance planning is also defined. For the VR interface, real-time environment and maintenance is prioritized. The user interface integrates GUI, interaction, and visual display.
- The 3D VR environment focuses on the seamless integration of technologies and tools. The VR environment is built using Unreal Engine and the HTC Vive system with Headset and Interaction devices.
- To visualize the maintenance and repair, the VR demo focused on a target company. The requirements were gathered and identified as use cases for the user to interact with. The use cases included actions for movement and interaction of the user with the system to perform maintenance and repair actions. A mini-map enables the user to navigate. These use cases act as the requirements for building the system.
- The VR focuses on industrial maintenance and performs the operations and maintenance of a power plant. The control room for the power plant and a dashboard serves as a monitoring interface for the status and to highlight faults that need attention. Tasks, for example, could be to identify leaks, turn on/off the valves, or to replace filters.
- The empirical and managerial implications deal with providing quality customer service. This is done by employing several service engineers and providing them quality training both in VR and physical operations. The VR environment is reported to have served quality training to the new service engineers.

**Fig. 12:**
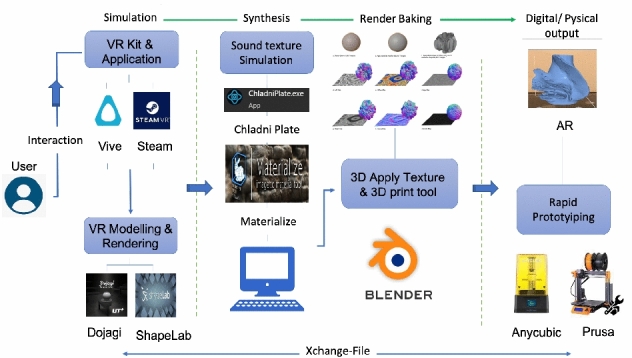
Functionality of VP system

**Fig. 13:**
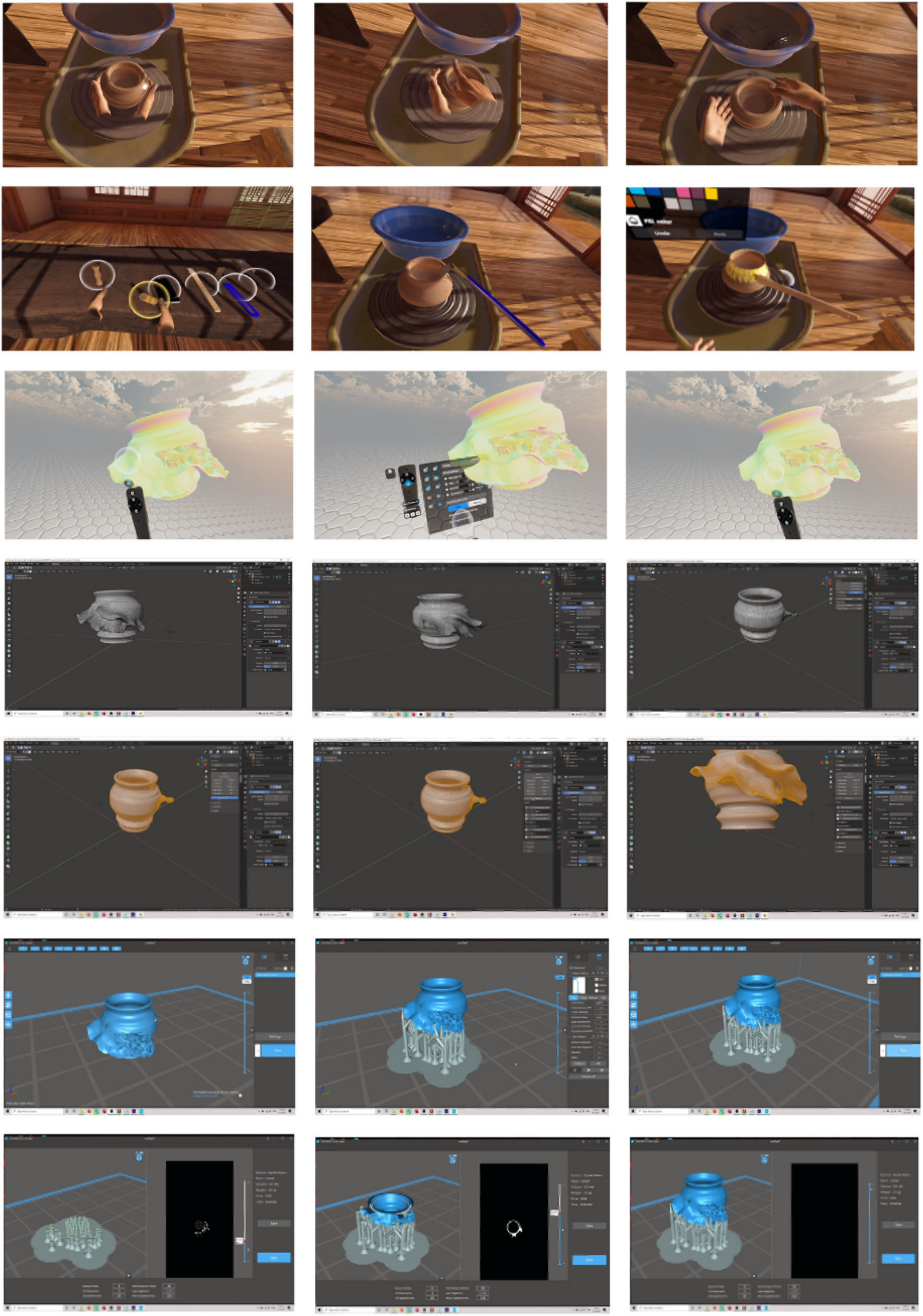
VP system shown in keyframes. The keyframes show the forming (top rows), sculpting (3rd row), adding surface relief (4th row) and the preparation for fabrication (last two rows)

In contrast, Dashti et al. [60] proposes a set of stages of experimental approaches and presenting evaluations of VP systems, extending the functionality of the system with usability development shown in Fig. 12. The key stage is VR Modeling and Rendering using publicly available tools such as Dojagi and ShapeLab. For user interaction, the VR Kit and software used are Vive and Steam. An independent stage is the computer Synthesis of surface details. The sound texture is simulated using Chladni Plate software and Materialize software. The next step is to integrate the simulated 3D Pottery and the synthesized texture to bake the 3D detailed shape model, which is done using the Blender software. Our approach differs from the above two demonstration prototypes in the data physicalization phase. The sound resonance data is physicalized along with the VR shape model. Extensive visualization helps in identifying potential errors in 3D printing. Anycubic and Prusa 3D printing, associated software, and materials are used for physical realization. The VP system has the potential to evolve further to support collaboration as well as to have a fully integrated digital environment, and we show the schematic in Fig. 11. We have also included a video demo of our system in action. Keyframes of the video are shown in a tabulated form in Fig. 13. Figure 13 shows the process of our system, (a) in the top two rows for shape modeling using the Dogagi tool for basic shape modeling and sculpting, (b) the third row shows the use of the Shapelab app for sculpting and analyzing objects, (c) fourth and fifth row uses Blender for applying texture, and retopology wit objects analysis, (d) The sixth and seventh row uses Chitubox to prepare for 3D printing.

**Fig. 14:**
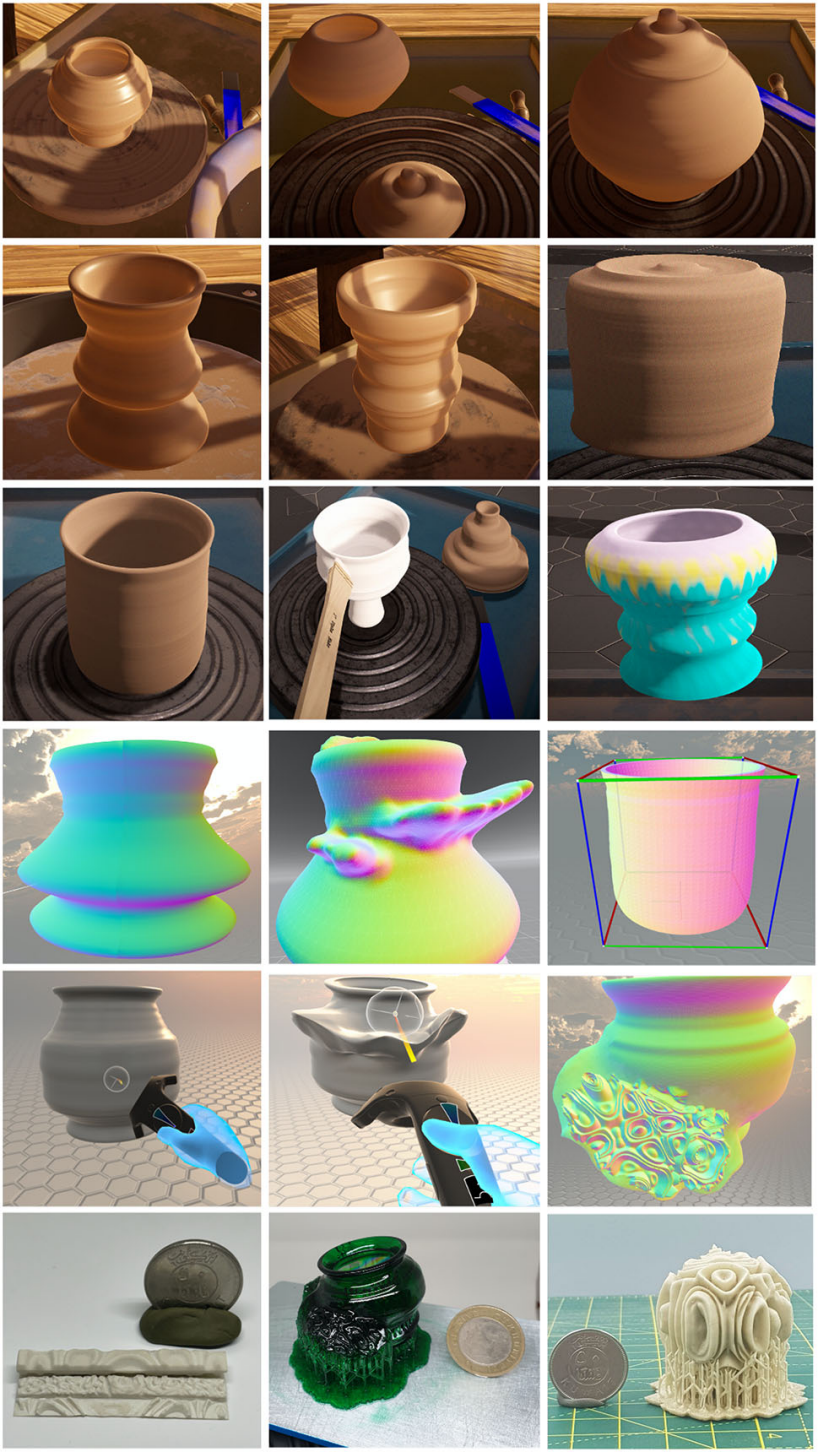
Experimental results of VP modeling and sculpting in the first three rows: (1–3) the process of developing VP pottery basic modeling on VR wheel with sensitive haptic feedback in and out of the object, (4) extending with freeform modeling and sculpting, (5) texture application and viewing in VR, (6) and finally prototyping with ceramic resin

In Fig. 14 we show our experimental results of Virtual and Physical Pottery Models VR modeling (in top two rows) on VR wheel creating the basic VP model. Then extending the VP model with freeform modeling using sculpting VR tools sculpting with ability to view and deform the 3D texture in VR application, 3D visualization (middle row), interaction and mesh blending (row 4), 3D printing (bottom row)].

Our system is comparable to the two systems presented here and is similar in terms of the framework, except they target different applications. The systems have a basic shape model used to build the VR environment but overlay simulation data to see some physical behavior that changes the basic environment, such as shape deformations, reactions visualization, or change in surface shape detail. There is no single software, technology, or tool today to build a complete system. Hence, the VR applications need to tie-up multiple software and hardware tools together to realize a VR application. While an attempt is made to integrate and realize a seamless system, the nature of the simulation dictates the level of integration. Hence, the integration is achieved through data exchange between the systems.

This also facilitates social collaboration, as the VP users can collaborate from any networked location across the globe. We show the interplay between the virtual and physical, in our case through 3D rapid prototyping/printing (see Table 2), which is possible through accessible 3D printers. The VP system goes beyond what is done in the physical space. It enhances the capability of the VP learner or user, as the virtual prototype and the physical prototype can be visualized in much less time, than in physical pottery. The VP allows to save snapshots and helps to restore to previous states and recreate the repetitive process easily, instead of starting from scratch every time a damage is done to the model. The example including the 3D printed prototype shown in Fig. 3 was done in less than a day, and this can be accomplished once the user has sufficient training and gained skills to use the system.

## Conclusion

We have demonstrated the design and development of our novel PotteryVR system. We presented an intensive literature review to explain the concept of our system and how the user interaction and usability factors can assist in extending VP systems. The provided comparison tables of VP systems explain the development aspects for VP/3D modeling advancement. The modeling outputs in Fig. 12 of our system shows our experiments of the traditional pottery essence, creating more realistic organics forms with the representation of visual and physical texturing outputs with the prototype. The VP system is now scalable, as it is not restricted to a physical space. Several instances of the VP system can be launched simultaneously. The tools that we have used to demonstrate the system uses subsystems that are not expensive and affordable. We believe our VP system will have wider adoption with more affordable creative tools and technologies. Adding multiple users in the system is also feasible now through the VE and users need not occupy the same physical space.

Moving on, we focused on the usability evaluation methods based on research studies related to the field of VR, discussing the evaluation methods that will advance our research based on Visual Feedback, Gestural Interfaces, 3D modeling, and Collaborative Design. The feedback focuses on the usability of integrating multi-disciplinary VR and creative technologies, relying mainly on visual feedback and image-based studies of VR applications. This approach optimizes the outcomes in terms of our novel VP system, configuring traditional pottery modeling, utilizing a combination of virtual, 3D modeling, and fabrication tools for interactive quality and dynamic compatibility. The usability studies aim to develop modeling and practices skills, providing guidelines on designing more solid applications, employing VP as an extended version of deformable shape modeling, and rapid prototyping. Refinements in hand tracking and gestural interfaces show great promise [61], and this could be evaluated for PotteryVR as part of future research. Human factors are essential to VE, as demonstrated in recent research results [62, 63] and the human factors could be evaluated for PotteryVR. A possible direction for future work would be to expand our system to a fully integrated Collaborative PotteryVR system.

## Declarations

### Human or animals rights

The authors declare that this research does not involve human participants or animals.
